# Development of a combination of noradrenergic and antimuscarinic drugs for the treatment of obstructive sleep apnea: Challenges and progress

**DOI:** 10.3389/frsle.2023.1148282

**Published:** 2023-03-03

**Authors:** Luigi Taranto-Montemurro, Huy Pho, David P. White

**Affiliations:** ^1^Apnimed Inc., Cambridge, MA, United States; ^2^Brigham and Women's Hospital, Boston, MA, United States

**Keywords:** combination therapy for OSA, pharmacotherapy for OSA, norepinephrine reuptake inhibitors, antimuscarinics, ato-oxy

## Abstract

Obstructive sleep apnea (OSA) is a disorder characterized by repetitive collapse of the upper airway during sleep, leading to intermittent hypoxia and sleep fragmentation. The combination of noradrenergic and antimuscarinic drugs has emerged as a potential pharmacological treatment option for OSA, with the most promising combination being atomoxetine plus aroxybutynin. This combination is currently undergoing extensive experimentation and will be soon tested in phase 3 studies. Other noradrenergic drugs including reboxetine, and other antimuscarinics including fesoterodine, hyoscine butylbromide, solifenacin, and biperiden have been tested. The increasing interest in OSA pharmacotherapy is driven by advances in our understanding of the pathophysiology of the disease and accumulating evidence of the surprising effectiveness of this drug combination. However, challenges remain in accurately measuring the severity of OSA, which can impact our ability to fully understand the efficacy of these medications. Further research is ongoing to address these challenges and to optimize the use of noradrenergic and antimuscarinic drugs for the treatment of OSA.

## Introduction

To date, the search for a pharmacotherapy to treat the underlying cause of obstructive sleep apnea (OSA), i.e., the narrowing and obstruction of the upper airway during sleep, has been largely limited to small observational studies or proof-of-concept, short-term clinical trials mostly performed in academic settings (Taranto-Montemurro et al., 2019b). While these studies show occasionally encouraging results, often they are underpowered to detect an effect on OSA severity and even the positive study results can be difficult to replicate in subsequent clinical trials (Marshall et al., 2008). For these reasons, investigators have been reluctant to test drugs for OSA in large and expensive phase 2 or 3 trials.

The therapeutic space in OSA is largely dominated by continuous positive airway pressure (CPAP) (Sutherland et al., 2018). However, multiple recent trials showed that, due mostly to limited compliance, CPAP is not as effective as thought in treating OSA and in preventing adverse cardiovascular and neurocognitive outcomes (Kushida et al., 2012; Mcevoy et al., 2016). This fact has reinvigorated the research for alternative treatments for OSA. Moreover, recent developments in the understanding of OSA pathophysiology (Wellman et al., 2013) and the identification in animal models of potential targets for OSA pharmacotherapy (Horner et al., 2017) have generated new interest by pharmaceutical companies in this disorder. This has led to an increased number of large ongoing phase 2 and 3 trials (Clinicaltrials.gov, 2022a,b,c), which will hopefully advance the field of OSA pharmacotherapy in future years.

In this short review we will focus on the ongoing development of combinations of noradrenergic and antimuscarinic drugs for the treatment of OSA. Only published data from peer-reviewed journals will be reported and discussed.

## Recent discoveries in animal model

It has been known for many years (Remmers et al., 1978) that falling pharyngeal dilator muscle activity during sleep is one of the principle causes of OSA. However, elucidating the neural mechanisms underpinning this loss of muscle activity has required more modern scientific techniques. Many studies over the years have demonstrated that cells producing excitatory neurotransmitters such as serotonin and norepinephrine decrease their firing frequency during NREM sleep with further reductions during REM sleep (Aston-Jones and Bloom, 1981). In addition, the REM-related broad inhibition of skeletal muscle activity has been shown to result from active inhibition from glycine and GABA (Chase et al., 1989).

Recently, Richard Horner's lab in Toronto developed a rat preparation whereby natural sleep could be monitored using standard techniques, a microdialysis catheter placed in the hypoglossal motor nucleus could both measure and administer neurotransmitters/drugs, and genioglossal EMG (EMGgg) could be continuously recorded. Using this preparation, they first demonstrated that loss of genioglossal muscle activity during NREM sleep was primarily a product of reduced norepinephrine activation of the muscle, a disfacilitation mechanism (Chan et al., 2006). The application of an alpha agonist at the 12th motor nucleus during NREM sleep could largely restore muscle activity in rats. Previous work had suggested that reductions in serotonergic neural input to the genioglossus were most important in mediating sleep-related loss of muscle activity (Fenik et al., 2005). These previous findings led to numerous studies assessing the impact of medications to modify neural serotonin on OSA severity without great efficacy (Taranto-Montemurro et al., 2019b). However, it was later discovered that cutting the vagus nerve may have overemphasized the role of serotonin in regulating the genioglossus (Sood et al., 2005). Nevertheless, some investigators still believe serotonin may have a role in the upper airway muscle activation despite the failure of most such interventions to improve sleep disordered breathing (Kubin, 2016).

Although, as stated above, glycine and GABA are the primary inhibitors of skeletal muscle activity during REM sleep, this REM sleep mechanism is less clear for upper airway dilators muscles. Some data suggest that antagonists to glycine and GABA have little effect on pharyngeal muscle activity during REM sleep (Park et al., 2008). Further work in the Horner lab reported that a potentially important source of falling EMGgg during REM sleep could be active cholinergic (muscarinic) inhibition (Grace et al., 2013). Their application of the antimuscarinic agent scopolamine could largely restore genioglossal muscle activity in rats during REM sleep. Thus muscarinic inhibition may be more important than such inhibition by glycine or GABA in mediating REM sleep loss of pharyngeal dilator muscle activity.

Two unrelated mechanisms may each be contributing to falling pharyngeal dilator muscle activity during sleep, one during NREM and the other during REM sleep. Countering both falling norepinephrine levels during NREM sleep and increased muscarinic inhibition during REM sleep with an oral pharmacological agent may treat sleep apnea. A novel combination of atomoxetine, a selective norepinephrine reuptake inhibitor (SNRI) approved in the US for treating attention deficit/hyperactivity disorder, with oxybutynin, an antimuscarinic approved in the US for treating overactive bladder, has recently been studied for treatment of OSA.

## Proof-of-concept clinical trials

**Figure 1:**
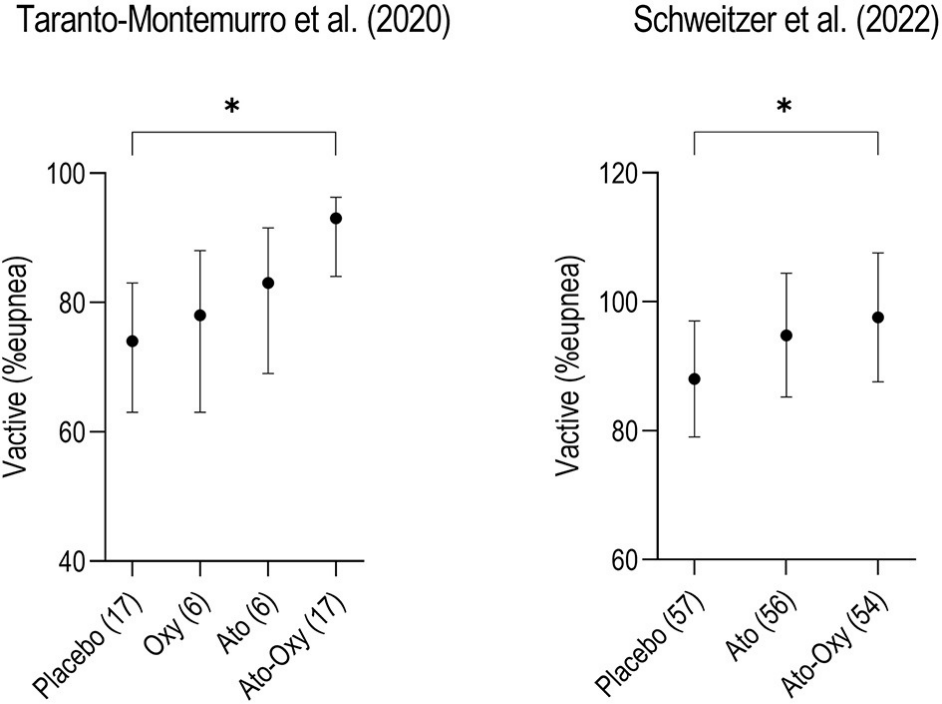
Two crossover trials comparing “active” upper airway collapsibility measured as Vactive between placebo, atomoxetine (ato) and atomoxetine plus oxybutynin (ato-oxy) showed that only the combination of drugs significantly increased Vactive compared to placebo, suggesting a synergistic effect of the combination on upper airway muscle activity. Vactive represents the ventilation measured at the arousal threshold, when the upper airway dilator muscles are maximally activated during sleep, just before the arousal. The same pattern is confirmed in another unpublished study with atomoxetine alone vs. AD109 (a combination of atomoxetine and R-oxybutynin, NCT04631107). Although the contribution of oxybutynin (oxy) in recruiting the pharyngeal muscles seems to be inferior compared to atomoxetine, it seems to have an important action in maximizing upper airway patency. Numbers in parenthesis on the x-axis represent the patients studied in each arm. Data for this figure show means (95% CI) and are taken from Taranto-Montemurro et al. (2020) and Schweitzer et al. (2022). *p < 0.05.

Table 1 provides a summary of the proof-of-concept randomized-controlled trials testing the combination of an SNRI and an antimuscarinic. A first trial performed at the Brigham and Women's Hospital in Boston (Taranto-Montemurro et al., 2019a) showed that the combination of atomoxetine and oxybutynin (ato-oxy) at doses of 80 and 5 mg, respectively, led to a clinically meaningful reduction in OSA severity in a group of 20 unselected patients. The reduction in AHI was associated with a ~3-fold increase in genioglossus muscle activity (measured using intramuscular electromyography). Additionally, in a subset of nine patients who returned to perform polysomnography for two subsequent nights, the administration of either agent alone did not lead to an AHI reduction compared to placebo. A follow-up multi-center confirmatory trial (Schweitzer et al., 2022) validated the efficacy of ato-oxy 80/5 mg in a group of 62 patients with low upper airway collapsibility defined as a higher proportion of hypopneas compared to apneas and an average oxygen desaturation of < 8% with disordered breathing events. In this crossover trial the authors studied both ato-oxy and atomoxetine alone, showing that atomoxetine had similar effect as the combination in reducing AHI, oxygen desaturation index (ODI) and hypoxic burden (HB). However, contrary to ato-oxy, atomoxetine alone did not reduce the rate of respiratory arousals vs placebo and there was a trend for reduced total sleep time (−26 min) on atomoxetine alone compared to ato-oxy (*p* = 0.06). Oxybutynin's main role may be reducing the sleep disruptive effects of atomoxetine by attenuating its wake-promoting activity. The analysis of the endotypic traits in both ato-oxy trials indicated that, while atomoxetine alone seems to play the largest role in reducing airway obstruction when compared to oxybutynin alone, only the combination improved “active” upper airway collapsibility (collapsibility at maximum ventilatory drive during sleep, Vactive), suggesting that ato-oxy has a stronger effect than atomoxetine alone in recruiting the upper airway dilator muscles and improving ventilation (Figure 1).

**Table 1:** Summary of proof-of concept clinical trials testing combinations of selective norepinephrine reuptake inhibitors (SNRIs) and antimuscarinics.

**First author**	**Journal (year)**	**Intervention arms**	**N, type of study**	**AHI4**			**AHI3a**			**HB**		
				**Placebo**	**SNRI Alone**	**Combination SNRI** + **Antimuscarinic**	**Placebo**	**SNRI Alone**	**Combination SNRI** + **Antimuscarinic**	**Placebo**	**SNRI Alone**	**Combination SNRI** + **Antimuscarinic**
Taranto-Montemurro, Luigi	Am J Resp Crit Care Med (2019)	Placebo/Atomoxetine (80 mg) + Oxybutynin (5 mg)	*N* = 20 crossover, 1 night				28.5 (10.9 to 51.6)		7.5^***^ (2.4 to 18.6)			
Aishah, Atqiya	Journal of Applied Physiology (2021)	Placebo /Atomoxetine 80 mg + Solifenacin 5 mg/Atomoxetine 80 mg + Biperiden 2 mg	*N* = 11 Crossover, 1 night				46 ± 22.5		Ato+Sol 51.0 ± 21.4 Ato+ Bip 48.3 ± 23.6			
Lim, Richard	Journal of Physiology (2021)	Placebo/Reboxetine 4 mg + Hyoscine Butylbromide 20 mg	*N* = 12 Crossover, 1 night				51 ± 30		33 ± 22^**^			
Perger, Elisa	CHEST (2021)	Placebo/Reboxetine 4 mg + Oxybutynin 5 mg	*N* = 18 crossover, 1 week				38.7 (29.0 to 47.8)		18.0^***^ (12.5 to 21.4)	75.5 (68.1 to 168.0)		39.7^***^ (25.4 to 55.3)
Schweitzer, Paula K.	Sleep and Breathing (2022)	Placebo/Atomoxetine 80 mg/Atomoxetine 80 mg + Oxybutynin 5 mg	*N* = 62 Crossover, 1 night	14.2 (5.4 to 22.3)	4.8^***^ (1.4 to 11.6)	6.2^***^ (2.8 to 13.6)	23.6 (12.4 to 32.7)	15.4^***^ (9.0 to 27.9)	14^***^ (8.1 to 17.1)	30.5 (10.4 to 31.6)	9.7^***^ (3.3 to 28.8)	13.7^***^ (4.4 to 30.3)
Rosenberg, Russel	Journal of Clinical Sleep Medicine (2022)	Placebo/AD109 37.5/2.5/AD109 75/2.5	*N* = 31 crossover, 1 night	13.2 (8.0 to 19.1)		AD109 37.5/2.5 7.8^*^ (4.0 to 13.7) AD109 75/2.5 5.5^***^ (2.2 to 9.6)				13.9 (4.5 to 21.9)		AD109 37.5/2.5 7.3^**^ (2.0 to 12.5) AD109 75/2.5 2.3^***^ (0.1 to 10.5)
Messineo, Ludovico	Respirology (2022)	Placebo/Atomoxetine 80 mg + Fesoterodine 4 mg	*N* = 12 crossover, 1 night				34.2 ± 19.1		30.1 ± 28.2	52.4 ± 50.5		29.7 ± 78.9
Altree, Thomas J.	Journal of Clinical Sleep Medicine (2022)	Placebo/Reboxetine 4 mg/Reboxetine 4 mg + Oxybutynin 5 mg	*N* = 16 crossover, 1 night	18 ± 17	13 ± 16^*^	14 ± 17^*^	36 ± 15	31 ± 14^*^	32 ± 17	74 ± 60	56 ± 57	56 ± 50^*^
Aisha, Atqiya	Annals of American thoracic society	Placebo /Atomoxetine 80 mg + Oxybutynin 5 mg /Atomoxetine 40 mg + Oxybutynin 5 mg/Atomoxetine 40 mg + Oxybutynin 2.5 mg	*N* = 39 parallel arms, 30 nights	Placebo from 13 ± 12 to 13 ±15		Ato-Oxy 80/5 from 24 ± 18 to 12 ± 11 (*p < * 0.05 at Night 1) Ato-Oxy 40/5 from 22 ± 16 to 20 ± 16 Ato-Oxy 40/2.5 from 23 ± 24 to 19 ± 19^*^	Placebo from 29 ± 14 to 28 ± 16		Ato-Oxy 80/5 from 39 ± 19 to 29 ± 15 Ato-Oxy 40/5 from 35 ± 23 to 37 ± 18 Ato-Oxy 40/2.5 from 45 ± 24 to 42 ± 22	Placebo from 42 ± 52 to 40 ± 59		Ato-Oxy 80/5 from 84 ± 96 to 34 ± 41 (*p < * 0.05 at Night 1 and 30) Ato-Oxy 40/5 from 76 ± 64 to 70 ± 75 Ato-Oxy 40/2.5 from 77 ± 130 to 47 ± 54

Data are expressed as medians (IQR) or means ± SD. Only double blinded, placebo-controlled trials have been reported here. SNRI, selective norepinephrine reuptake inhibitor; AHI3a, apnea hypopnea index, 3% or arousal definition for hypopneas; AHI4, apnea hypopnea index, 4% definition for hypopneas; AD109, combination of atomoxetine + R-oxybutynin. ^*^p < 0.05, ^**^p < 0.01, ^***^p < 0.001 vs. placebo. The trials reported above excluded male patients >65 due to concerns for higher risk of urinary problems with ato-oxy or AD109 in the older male population. Women over 65 years of age were allowed in the trial without any reported safety concerns.

Aisha et al. assessed tolerability and safety of three doses of ato-oxy after 30 days of treatment in a placebo-controlled, parallel arms study (Aishah et al., 2022). In this small trial, which enrolled 39 patients across 4 treatment arms, the authors found that ato-oxy was well tolerated, with the most common side effects being dry mouth, dyspepsia and nausea. They also observed that only the high dose of ato-oxy, 80/5 mg, reduced the AHI on day 1 (*p* < 0.05) and on day 30 (*p* = 0.09) by ~50%. HB, a recently introduced OSA severity metric quantitively assessing the oxygen desaturation associated with upper airway obstructive events (Azarbarzin et al., 2018), was also dramatically reduced by >80% vs baseline at both timepoints (*p* < 0.01) with high dose ato-oxy. Interestingly, only when hypopneas were scored using the 4% desaturation criterion according to the American Academy of Sleep Medicine (AASM) alternative definition (AHI4), was there a statistically significant reduction in AHI comparable to previous findings. On the contrary, when hypopneas were scored in association with 3% desaturation or arousal (AHI3a) there was no significant effect of the combination on OSA severity.

In subsequent proof-of-concept studies aimed at identifying the effects of other antimuscarinics in combination with atomoxetine, fesoterodine (Messineo et al., 2022), solifenacin, and biperiden (Aishah et al., 2021) all had lesser efficacy than oxybutynin, possibly due to their more selective action on muscarinic receptors (solifenacin, biperiden). This also could be due to their lower permeability across the blood brain barrier compared to oxybutynin (fesoterodine). A recent study performed in 17 Japanese OSA patients showed no effect of ato-oxy on overall OSA severity, although a subset of patients experienced AHI reduction (Kinouchi et al., 2022). While this study suffers from methodological limitations such as lack of placebo and blinding, it suggests that ethnicity may play a role in the response to this combination.

Recent research efforts have addressed the development and efficacy of the combination of the R-enantiomer of oxybutynin (aroxybutynin) with atomoxetine (combination named AD109) with the goal of improving risk-benefit in OSA compared to the combination with racemic oxybutynin. Indeed, oxybutynin is commercially available in a racemic form composed of 50% S-oxybutynin and 50% R-oxybutynin (aroxybutynin). The efficacy of oxybutynin in OSA is believed to be related to its antimuscarinic effects. The R-enantiomer of oxybutynin has been shown to confer the antimuscarinic effect of oxybutynin, whereas the spasmolytic effects (and other effects on calcium channel antagonism and local anesthetic effects) are non-stereoselective properties of both the enantiomers (R and S). Most recently, Rosenberg et al. tested two doses of Apnimed's AD109 (37.5/2.5 and 75/2.5 mg of atomoxetine/aroxybutynin) during a crossover trial in patients with mild to moderately severe OSA [AHI4 between 5 and 20 events/h (Rosenberg et al., 2022)]. The combination showed at both doses a statistically significant reduction of AHI4 and HB compared to placebo after acute (1-night) administration. The study demonstrated a dose-response for AD109, with the effect size of high dose AD109 being larger than that of low dose AD109.

Another line of investigation aimed to test the effect of reboxetine, another SNRI, taken alone or in combination with an antimuscarinic, on OSA severity. During a crossover trial, Lim et al. successfully reduced the AHI by ~35% with reboxetine 4 mg and hyoscine butylbromide 20 mg administered for 1-night and showed an increase in genioglossus activity on drugs vs placebo (Lim et al., 2019). Perger et al. showed, in another crossover trial, that 1 week of reboxetine 4 mg plus oxybutynin 5 mg reduced OSA severity by ~60% (*p* < 0.001) (Perger et al., 2022).

Finally, Altree et al. recently tested, in a single night, randomized controlled crossover trial, the combination of reboxetine plus oxybutynin vs reboxetine alone vs placebo (Altree et al., 2022). Contrary to the previous experiments, the combination did not significantly reduce the AHI3a, while reboxetine alone showed an average AHI3a reduction of ~15% vs placebo (*p* = 0.03). As discussed above, the results were different depending on the hypopneas scoring criteria used. When AHI4 was assessed, both reboxetine alone and the combination with oxybutynin reduced OSA severity compared to placebo. Finally, as was observed with atomoxetine, there was a tendency for reboxetine alone to reduce total sleep time by ~20 mins and sleep efficiency by 6% (*p* = 0.11) compared to the combination with oxybutynin.

## Interpretation challenges of the proof-of-concept trials

The proof-of-concept trials mentioned above raised several interpretation challenges. The most important are related to (a) the mechanism of contribution of the antimuscarinics (racemic oxybutynin or aroxybutynin) to the combinations tested and (b) the reconciliation of variable results across multiple trials.

*a) The contribution of the antimuscarinics (racemic oxybutynin or aroxybutynin)*

The original hypothesis of the investigators was that the main role of oxybutynin was to enhance upper airway dilator muscles activity especially during REM sleep by blocking the muscarinic inhibitory pathway to genioglossus activation. However, it has become clear, after multiple similar findings, that the SNRIs (atomoxetine or reboxetine) have the most important stimulatory action on the pharyngeal dilator muscles, while the antimuscarinic component has a smaller such effect. The available data on the effect of oxybutynin taken alone indicate no specific reduction on REM AHI. A potential explanation for this occurrence might be that rather than a cholinergic mechanism becoming active only during REM sleep to inhibit hypoglossal motor activity, there might exist a constant cholinergic inhibition throughout all states (wake, NREM, and REM), but it is most noticeable in REM sleep due to the absence of inputs that support muscle activation, such as noradrenergic inputs. As a result, the loss of noradrenergic inputs plays a role in reducing muscle activity during non-REM sleep, while both the noradrenergic and cholinergic mechanisms contribute to motor suppression during REM sleep. It is unlikely that a single mechanism is responsible for motor suppression in each state, such as non-REM adrenergic inhibition and REM cholinergic inhibition. The contribution of oxybutynin to upper airway muscle stimulation is revealed by the synergistic effect, during NREM sleep, of the ato-oxy combination on the “active” upper airway collapsibility (Vactive, see Figure 1 for details) (Taranto-Montemurro et al., 2020). It is important to highlight that the analysis of REM data is limited by the acute reduction in REM sleep that is typically seen with SNRIs administration. The longest study (30 days) performed with ato-oxy suggests that a partial recovery of REM sleep is likely to occur after a few weeks of therapy (Aishah et al., 2022) and more data on REM sleep may be available with larger, long-term studies.

A second important contribution of the anticholinergic agent in the ato-oxy combination was discovered to be the mitigation of the wake-promoting effects caused by the SNRIs. Indeed, the monoaminergic and cholinergic systems are largely wake-promoting (Schwartz and Kilduff, 2015) with basal forebrain cholinergic neurons activating cortical pyramidal cells which augment cortical activation and EEG desynchronization (Sofroniew et al., 1982; Dunnett et al., 1991). Conversely, antimuscarinic medications have sedative properties (Thornton, 1977; Weerts et al., 2015) and this effect may be mediated by the reduction in basal forebrain cholinergic activation (Anaclet et al., 2015). Antimuscarinic drugs, such as atropine, have been found to eliminate the fast, low-amplitude brainwaves induced by adrenergic stimulants, such as amphetamine, in animal studies. Instead, these drugs lead to the development of slow, high-amplitude brainwaves that are characteristic of NREM sleep. In the context of OSA treatment, the combined effects of an antimuscarinic which increases pharyngeal muscle activity and improves sleep consolidation may be an ideal solution. Oxybutynin may also have less risk of next morning sedation or muscle relaxation compared to commonly prescribed hypnotics.

*b) Reconciliation of variable results across multiple trials*

As discussed above, not all the small trials to date involving a combination of SNRI and antimuscarinic have yielded similar results. Although it is not possible in this short review to provide a detailed discussion of all possible explanations for these different outcomes, there are quite a few possibilities. Among the possible reasons for this lack of reproducibility in proof-of-concept trials results are the different properties of drugs used from the same class, patient heterogeneity, methodological differences, and the spontaneous night-to-night variability in OSA severity. In addition, interscorer variability may cause different interpretation of the sleep studies, and the different definitions of AHI used at different institutions can importantly alter trial results. To address this last issue, according to the AASM criteria, hypopneas may be scored when associated with a 3% desaturation or arousal (AHI3a) or, alternatively, when associated with a 4% oxygen desaturation (AHI4) (Berry et al., 2012). While the second definition of hypopnea is more conservative [AHI4 may be >50% lower than AHI3a (Ruehland et al., 2009)], it also yields the greatest reproducibility across different scorers as it avoids the scoring of arousals in the determination of AHI. It has been clearly observed that arousals are the largest source of interscorer variability and definitions of AHI which include arousal scoring therefore result in less reproducibility across sites and across trials (Loredo et al., 1999). Other scoring criteria for hypopneas may vary from study to study including the required reduction in flow amplitude which may be 30% or 50% from baseline depending on definitions used (Ruehland et al., 2009). A solution to these inconsistent scoring rules could be the adoption of validated automatic scoring services which are increasing in number and quality. In addition, the search for new metrics that may better represent the real ventilatory deficit associated with upper airway obstruction has yielded the HB of OSA (Azarbarzin et al., 2018). This deficit is currently only partially captured by the AHI, which is a simple frequency metric with scarce correlation to clinical symptoms or long-term outcomes of OSA (Malhotra et al., 2021).

Some of these issues related to the diagnostic paradigm of OSA are being discussed by academic experts (Mehra et al., 2023) and have been recently considered while designing larger industry-sponsored trials testing the effects of pharmacotherapies on OSA severity (Clinicaltrials.gov, 2022a,c; Hedner et al., 2022). The Mariposa trial was a large 25-center phase 2b trial recently concluded, which tested the effect of AD109 over a month of therapy in patients with a baseline AHI4 between 10 and 45 events/h. To reduce the effect of night-to-night variability, the AHI was collected and averaged over 2 nights both at baseline and on treatment. This same strategy was used during the investigation of sulthiame, a new carbonic anhydrase inhibitor tested for OSA treatment from Hedner and colleagues. The use of two-night assessments may have played a role in reducing the amount of variability in individual responses compared to other studies on carbonic anhydrase inhibitors (Hedner et al., 2022). In Mariposa, AHI4 was selected as the primary outcome to increase the reproducibility of the results across trials and across scoring centers, and the HB was also quantified as in previous trials with the same therapy (Rosenberg et al., 2022). Longer trials are also exploring several patients reported outcomes. Subjective outcomes in OSA pharmacotherapy have been largely overlooked so far but are clearly important to fully understand the impact of treatment (Hedner and Zou, 2022).

## Conclusion

The use of a combination of selective norepinephrine reuptake inhibitors (SNRIs) and antimuscarinic drugs has shown promise in the search for a pharmacotherapy for OSA. While progress has been made in this area, there are still hurdles that need to be overcome in order to bring a treatment based on AD109 to patients. These include the need for larger and longer trials to better define both the subjective and objective outcomes of therapy with this drug combination. In addition, although the AHI is considered the gold standard for evaluating the presence and severity of OSA, there are clear limitations to its accuracy as a metric for measuring the extent of the breathing disorder and the effectiveness of treatments. This problem is still being addressed in ongoing research. Despite these challenges, the prospect of a pharmacotherapy for OSA is becoming increasingly promising, and further research and development in this area may bring us closer to a viable treatment option for this common and debilitating condition.

## Author contributions

LT-M contributed to data analysis and interpretation and drafting and review of the manuscript. DW contributed to data interpretation and drafting and review of the manuscript. HP contributed to data analysis and review of the manuscript. All authors contributed to the article and approved the submitted version.
